# Analysis of prognostic factors in 766 patients with small cell lung cancer (SCLC): the role of sex as a predictor for survival.

**DOI:** 10.1038/bjc.1991.215

**Published:** 1991-06

**Authors:** M. Wolf, R. Holle, K. Hans, P. Drings, K. Havemann

**Affiliations:** Department of Internal Medicine, Philipps-University of Marburg, Germany.

## Abstract

The data of 766 patients participating in three German multicentre trials were analysed with regard to the relationship between baseline characteristics and prognosis in small cell lung cancer (SCLC). The central aim of this analysis has been to evaluate the role of gender as an independent prognostic factor in SCLC. The minimum follow-up period for the 652 male and 114 female patients was 36 months. Female patients were shown to have a higher complete remission rate (35% vs 25%), a superior median survival (ms) (12.1 months (mo) vs 9.8 mo), and a favourable 2-year survival rate (2ys) (19% vs 8%) to male ones. Various other prognostic factors have been proved to be significant, such as extent of disease, clinical performance status, and history of smoking, whereas weight loss prior to chemotherapy and age have been less important factors. We have been able to ascertain that women's responses were better than those of male patients independent of any other relevant prognostic variable. Furthermore, results were found to be even more advantageous for female patients with additional favourable prognostic parameters, i.e. for patients with limited disease (ms 15.2 mo vs 12.0 mo; 2ys 29% vs 9%) or with good performance status (ms 13.4 mo vs 10.4 mo; 2ys 24% vs 7%). A most remarkable observation was made in that the favourable prognostic effect of the female gender was restricted to patients aged less than 60 years (ms 13.3 mo vs 10.1 mo; 2ys 26% vs 5%), whereas for older women no advantages over men's results were established (ms 9.3 ml vs 9.1 mo; 2ys 8% vs 7%). A proportion of 32% of female patients with limited disease aged less than 60 years achieved a 3-year survival rate. We conclude (a) that sex constitutes a major prognostic factor in SCLC and is especially useful as a predictor for long-term survival, and (b) that the favourable prognostic value of the female sex is restricted to younger patients.


Br. J. Cancer (1991), 63, 986–992 © Macmillan Press Ltd., 1991

### Analysis of prognostic factors in 766 patients with small cell lung cancer (SCLC): The role of sex as a predictor for survival

M. Wolf[1], R. Holle[2], K. Hans[3], P. Drings[4] & K. Havemann[1]

[1]*Department of Internal Medicine, Division of Hematology/Oncology, Philipps-University of Marburg;* [2]*Biostatistics and Data Centre (ZMBT), University of Heidelberg;* [3]*Evangelische und Johanniter-Krankenanstalten, Oberhausen;* [4]*Krankenhaus Rohrbach, Thoraxklinik der LVA Baden, Heidelberg, Germany.*

**Summary** The data of 766 patients participating in three German multicentre trials were analysed with regard to the relationship between baseline characteristics and prognosis in small cell lung cancer (SCLC). The central aim of this analysis has been to evaluate the role of gender as an independent prognostic factor in SCLC. The minimum follow-up period for the 652 male and 114 female patients was 36 months. Female patients were shown to have a higher complete remission rate (35% *vs* 25%), a superior median survival (ms) (12.1 months (mo) *vs* 9.8 mo), and a favourable 2-year survival rate (2ys) (19% *vs* 8%) to male ones. Various other prognostic factors have been proved to be significant, such as extent of disease, clinical performance status, and history of smoking, whereas weight loss prior to chemotherapy and age have been less important factors. We have been able to ascertain that women's responses were better than those of male patients independent of any other relevant prognostic variable. Furthermore, results were found to be even more advantageous for female patients with additional favourable prognostic parameters, i.e. for patients with limited disease (ms 15.2 mo *vs* 12.0 mo; 2ys 29% *vs* 9%) or with good performance status (ms 13.4 mo *vs* 10.4 mo; 2ys 24% *vs* 7%). A most remarkable observation was made in that the favourable prognostic effect of the female gender was restricted to patients aged less than 60 years (ms 13.3 mo *vs* 10.1 mo; 2ys 26% *vs* 5%), whereas for older women no advantages over men's results were established (ms 9.3 ml *vs* 9.1 mo; 2ys 8% *vs* 7%). A proportion of 32% of female patients with limited disease aged less than 60 years achieved a 3-year survival rate. We conclude (a) that sex constitutes a major prognostic factor in SCLC and is especially useful as a predictor for long-term survival, and (b) that the favourable prognostic value of the female sex is restricted to younger patients.

Small cell lung cancer treatment during the last decade has seen no notable alterations, with an average median survival of one year and a stable proportion of 5% to 10% of 2-year-survivors among large patient populations (Minna *et al.*, 1985; Havemann *et al.*, 1987*a*; Wolf *et al.*, 1987; Havemann *et al.*, 1987*b*) although efforts have been launched worldwide in order to increase the activity of chemotherapy regimens and a significant progress in the understanding of tumour biology has been made. With regard to achieving further improvement of survival rates, approaches aiming at variations in chemotherapy schedules seem to be less promising. Thus the main challenges in treatment of SCLC will be found in (a) the creation of completely new treatment modalities based on experimental studies on tumour biology, and (b) the introduction of individualised treatment modalities for subsets of patients with differing prognoses. For these aims to be achieved, extensive evaluations of prognostic variables in SCLC will have to be made available. Up until now only two variables out of a variety of patients' baseline characteristics, namely extent of disease and clinical performance status, have been identified as consistent factors with an essential value for survival prognosis (Morstyn *et al.*, 1984; Livingston *et al.*, 1978; Osterlind *et al.*, 1986; Souhami *et al.*, 1985; Kalter *et al.*, 1984; Vogelsang *et al.*, 1985; Ihde *et al.*, 1981; Rawson *et al.*, 1990). Data about the prognostic value of the patient's sex have been controversial. Several authors maintain that the sex of the patient lacks significant prognostic impact (Souhami *et al.*, 1985; Ihde *et al.*, 1981; Rawson *et al.*, 1990; Einhorn *et al.*, 1978; Vincent *et al.*, 1987), whereas others have been able to show a superior outcome for female patients for subgroups of patients at least (Osterlind *et al.*, 1986; Maurer *et al.*, 1981; Sagman *et al.*, 1988; Dearing *et al.*, 1988; Spiegelman *et al.*, 1989). Therefore, the major aim of this investigation has been to analyse the prognostic value of sex of the patient in a large series of individuals, and to define its impact on survival prognosis.

### Patients and methods

From May, 1981, to December, 1986, a total of 784 patients with SCLC were accrued by 15 hospitals across Germany who then participated in three consecutive multicentre randomised trials.

Criteria for eligibility, staging, stratification and reevaluation were the same in all studies and have already been described (Havemann *et al.*, 1987*a*; Wolf *et al.*, 1987; Havemann *et al.*, 1987*b*). All studies were to be effectuated as randomised trials, the schedules of which are given in detail in Table I.

In study no. I, a comparison was made between the sequential CAV therapy and an alternating therapy with three different drug combinations (EVI/PAV/CMC) (Havemann *et al.*, 1987*a*). In study no. II, an ifosfamide/etoposide (IE) chemotherapy and a cisplatinum/etoposide (PE) chemotherapy were compared (Wolf *et al.*, 1987). In study no. III, a fixed cyclic alternating chemotherapy with IE and CAV was contrasted to a response-orientated alternating chemotherapy with IE therapy up to the maximum response and a subsequent switch to CAV following immediately afterwards (Havemann *et al.*, 1987*b*).

In each study, patients with limited disease received chest irradiation after the chemotherapy had been completed. Forty-five Gy were given in 23 fractions over 5 weeks. Prophylactic cranial irradiation was administered to all patients with complete remission. Thirty Gy were given in ten fractions within 2 weeks following the third cycle of chemo-

Correspondence: M. Wolf, Philipps-University of Marburg, Department of Internal Medicine, Division of Hematology/Oncology, Baldingerstrasse, D-3550 Marburg, Germany.
Participating institutions of the multicenter trials: Zentrum für Innere Medizin, Marburg; Krankenhaus Rohrbach, Heidelberg; Evangelische und Johanniter-Krankenanstalten, Oberhausen; St Johannes-Hospital, Duisburg; Klinikum, Mannheim; Städtische Kliniken, Fulda; St Elisabethen-Krankenhaus, Ravensburg; Krankenhaus an der Lieth, Bovenden; Medizinische Klinik, Kiel; Zentrum für Innere Medizin, Giessen; Fachklinik Schillerhöhe, Gerlingen; Kreiskrankenhaus, Mayen; Stadtkrankenhaus, Passau; St Barbara-Hospital, Gladbeck.
Received 10 April 1990; and in revised form 10 January 1991.

**Table I** Treatment regimens for three consecutive trials

| | | | |
|---|---|---|---|
| *Study I* | *Arm A:* cyclosphamide | 1000 mg/m$^2$ i.v. on day 1 | |
| | adriamycin | 50 mg/m$^2$ i.v. on day 1 | |
| | vincristine | 2 mg i.v. on day 1 | |
| | (8 cycles in 3-week intervals. Non-responders were switched to the arm B regimen.) | | |
| | *Arm B:* etoposide | 80 mg/m$^2$ i.v. on days 1–3 | |
| | vindesine | 3 mg/m$^2$ i.v. on days 1 | |
| | ifosfamide | 1500 mg/m$^2$ i.v. on days 1–5 | |
| | (cycles 1, 3 and 5) alternating with | | |
| | cisplatinum | 90 mg/m$^2$ i.v. on day 1 | |
| | adriamycin | 60 mg/m$^2$ i.v. on day 1 | |
| | vincristine | 2 mg i.v. on day 1 | |
| | (cycles 2, 4 and 6) and followed by | | |
| | cyclophosphamide | 1000 mg/m$^2$ i.v. on days 1 + 22 | |
| | methotrexate | 15 mg/m$^2$ p.o. on days 1,4,8,11 | |
| | CCNU | 100 mg/m$^2$ p.o. on day 1 | |
| | (Non-responders were switched to CAV.) | | |
| *Study II* | *Arm A:* cisplatinum | 80 mg/m$^2$ i.v. on day 1 | |
| | etoposide | 150 mg/m$^2$ i.v. on days 3–5 | |
| | (6 cycles in 3-week intervals. Non-responders were switched to CAV.) | | |
| | *Arm B:* ifosfamide | 1500 mg/m$^2$ i.v. on days 1–5 | |
| | etoposide | 120 mg/m$^2$ i.v. on days 3–5 | |
| | (6 cycles in 3-week intervals. Non-responders were switched to CAV.) | | |
| *Study III* | *Arm A:* ifosfamide | 1500 mg/m$^2$ i.v. on days 1–5 | |
| | etoposide | 120 mg/m$^2$ i.v. on days 3–5 | |
| | alternating with | | |
| | cyclophosphamide | 600 mg/m$^2$ i.v. on days 1 + 2 | |
| | adriamycin | 50 mg/m$^2$ i.v. on day 1 | |
| | vincristine | 2 mg i.v. on day 1 | |
| | *Arm B:* ifosfamide/etoposide therapy up to the maximum response and subsequently an immediate switch to cyclo-phosphamide/adriamycin/vincristine | | |

therapy or in the case of complete remission, on the onset of the same. No maintenance therapy was given to patients who had achieved complete remission.

### *Statistical methods*

Survival curves were calculated and plotted according to the Kaplan-Meier method. The statistical comparison of the survival curves for male and female patients was based on the log rank test (Peto *et al.*, 1977) with stratification for the two major prognostic variables, i.e. extent of disease and performance status unless stated otherwise.

An attempt of a comparative analysis of the relative influence of differing prognostic variables has been made by Cox's Proportional Hazard Model (Cox, 1972). Any analysis seeking to identify prognostic factors will have to be considered an exploratory effort, since it is impossible to determine beforehand all hypotheses that will be examined. Consequently the given $P$-values have not been corrected in terms of the effect of multiple testing on the error of the first kind and should be understood to be merely descriptive. Nevertheless, $P$-values of less than 0.001 can clearly be interpreted to signify statistical evidence of the prognostic value of a variable.

### Results

During the recruitment period, a total of 785 patients entered the three multicentre trials. Nineteen patients had to be excluded because of wrong histology (seven patients), surgical treatment prior to chemotherapy (two patients), or missing data (ten patients). Patient recruitment was stopped in December, 1986; the minimum follow-up time accordingly totals 36 months for 766 evaluable patients.

### *Analysis of survival according to treatment*

The three consecutive randomised trials showed very similar overall treatment results with a median survival of 10.6 months and a 2-year survival for a proportion of 10% of all patients. Since no significant differences were observed between the survival curves of each of the three trials, the entire number of 766 participating patients was then adopted as a basis for this study, and accordingly a combined analysis of prognostic factors of the whole group was undertaken.

### *The role of sex as an independent prognostic factor*

In each trial, the baseline characteristics age, sex, extent of disease, performance status, history of smoking, and weight loss prior to chemotherapy were recorded and subsequently included into a univariate analysis in order to determine the prognostic value of each variable. As shown in Table II, these five characteristics sex, extent of disease, performance status, history of smoking and weight loss prior to chemotherapy, were identified as the main prognostic factors, whereas age per se had no or only marginal impact on survival. It is well known that extent of disease and performance status are independent prognostic factors, while the effect of weight loss prior to chemotherapy depends to a large extent on the correlation with these two factors (cf. Table III which is explained below). Besides the extent of the disease and the performance status, the history of smoking was a third significant prognostic variable. With regard to the patient's sex, we noticed that female gender had an impressive advantage. Out of 114 females, 35% achieved a complete remission, median survival figured 12.1 months and a 2-year survival was achieved by 19%. On the other hand, the complete remission rate of the 652 male patients was 25%, median survival was 9.8 months, and a 2-year survival reached by 8%. The survival curves for both male and female patients are illustrated in Figure 1.

In concentrating our study in its second stage on the specific baseline characteristic sex, the next main task was to analyse whether the female gender constitutes an independent positive prognostic factor in small cell lung cancer. The stratified log rank test is well suited to determine the prognostic relevance of a certain characteristic when influences of a few other well known prognostic parameters can be elim-

**Table II** Relationship of baseline characteristics and survival

| | No. | CR + PR | CR | Median survival (months) | 2-year survival rate |
|---|---|---|---|---|---|
| *Sex* | | | | | |
| Males | 652 | 66% | 25% | 9.8 | 8% |
| Females | 114 | 69% | 35% | 12.1 | 19% |
| *Performance status* | | | | | |
| Karnofsky 50–70 | 154 | 48% | 12% | 6.2 | 2% |
| Karnofsky 80–100 | 606 | 72% | 30% | 10.8 | 11% |
| *Extent of disease* | | | | | |
| Limited | 294 | 79% | 39% | 12.5 | 14% |
| Extensive | 463 | 59% | 18% | 8.9 | 4% |
| *History of smoking* | | | | | |
| Smokers | 694 | 67% | 26% | 9.9 | 7% |
| Non-smokers | 63 | 65% | 30% | 13.6 | 17% |
| *Weight loss* | | | | | |
| Less than 10% | 638 | 69% | 29% | 10.5 | 7% |
| More than 10% | 112 | 54% | 13% | 8.3 | 4% |
| *Age* | | | | | |
| Less than 50 years | 156 | 68% | 30% | 11.2 | 8% |
| 50–60 years | 312 | 67% | 26% | 10.1 | 9% |
| More than 60 years | 298 | 64% | 24% | 9.2 | 7% |

No. = number of patients; CR = complete remission rate; PR = partial remission rate.

**Figure 1** Survival curve males *vs* females; (—) males, $n = 652$, median survival: 9.8 months; (---) females, $n = 114$, median survival: 12.1 months; $P = 0.0001$ (stratified log rank test).

inated. Thus the patients were stratified in terms of the two major prognostic factors upon which all investigators agree, namely stage of disease and performance status. In our univariate evaluation, the history of smoking was identified to be an additional prognostic variable. Initially this variable had not been included in the stratification design, because its prognostic relevance is not generally accepted, furthermore its inclusion would lead to the splitting up of the total number of patients into small subgroups thus limiting the reliability of the results. Using the stratified log rank test as described the survival of men and women differed significantly as the statistical results confirm with a $P$-value of 0.0001. Seeking a confirmation of this result and wishing to exclude any major influence of smoking, we repeated the stratified log rank tesk this time including also the history of smoking in the stratification design. The result found was nearly indentical with a $P$-value of 0.0002.

In a third stage of analyses, the prognostic value of the baseline characteristics was evaluated by using the Cox proportional hazard model. We have taken into account for the fact that the use of this test is connected with several statistical assumptions which are often ignored. A major problem is the assumed proportionality of the hazard functions which may require a re-scaling of prognostic variables or a stratified analysis. In the latter case, the stratification variable cannot be compared to other predictors in terms of its prognostic value, i.e. as far as our data are concerned, tests for proportionality of the baseline characteristics indicate that the two variables performance status and sex at least in part do not fullfil this fundamental requirement for an adequate use of the Cox model. The impact of the performance status on survival seems to be more pronounced in the early stages of the treatment course, whereas sex may be especially important for long-term survival. However, in spite of these effects, we have been able to confirm with the help of the Cox proportional hazard model that the patient's sex is essentially relevant for prognosis. The results of this analysis as shown in Table III indicate that the four variables sex, extent of disease, performance status and history of smoking constitute independent prognostic factors, whereas weight loss prior to chemotherapy and age have been found to be less important. The regression coefficients illustrate that the factors sex, performance status and history of smoking have had an almost indentical impact on the hazard function, i.e. on the patient's risk of dying. This influence was only surpassed by the impact by which extent of disease has on the prognosis.

*Differences in prognosis between male and female patients in important subgroups of patients*

The aim of the next step of the analysis was to find out, whether the influence of sex on the prognosis would prove to be homogeneous within all subgroups of patients. These were defined according to the extent of the disease, performance status, history of smoking, weight loss, and age. The results are summarised in Table IV.

*Extent of disease* The favourable outcome of female patients has been proved for those at a limited stage as well as for those at an extensive stage. Female patients with limited disease have been found to have a higher CR rate (44% *vs* 37%), a longer median survival (15.2 months *vs* 12.0 months), and in particular a higher 2-year survival rate (29% *vs* 11%) than males. This difference in survival is statistically significant ($P = 0.0015$, log rank test stratified by performance status). At an extensive stage, a better prognosis for women was noted, with a median survival of 10.2 months and a 2-year survival rate for 9% compared to 8.7 months and 7% for men. Although this difference seems to be less impressive, it is still highly statistically significant ($P = 0.0005$, log rank test, stratified by performance status).

*Performance status* Nearly all patients with a low performance status graded according to the Karnofsky index to 50–70% had a poor outcome. Although a longer median survival was achieved by female patients (9.0 months *vs* 5.9 months), the probability of long-term survival was minimal in both groups. In contrast to these results, in the subgroup of patients with a good performance status, a striking difference in prognosis has been seen. Female patients have achieved higher response rates (CR rate 39% *vs* 28%), longer median survival rates (13.4 months *vs* 10.4 months), and in particular a higher 2-year survival rate (24% *vs* 7%). The advantage of the female sex was statistically significant with a $P$-value of 0.0001 (log rank test, stratified by extent of disease).

*History of smoking* A superior survival rate for female patients was notable for smokers as well as for non-smokers. Non-smoking women had a favourable outcome with a

**Table III** Results of the first analysis by proportional hazard model*

| | Coefficient | Standard error | P-value |
|---|---|---|---|
| Sex | 0.40 | 0.11 | 0.0003 |
| Performance status | 0.45 | 0.10 | 0.0001 |
| Extent of disease | 0.61 | 0.08 | 0.0001 |
| History of smoking | 0.44 | 0.15 | 0.0026 |
| Weight loss | 0.19 | 0.10 | 0.071 |
| Age | 0.11 | 0.08 | 0.17 |

*The columns of the table give the estimated regression coefficient for the corresponding dichotomous variable in the Cox model, its standard error and the $P$-value for the test of the hypothesis that the coefficient equals zero.

**Table IV** Prognosis of females and males in important subgroups

| | No. | | Complete remission | | Median survival | | 2-year survival rate | |
|---|---|---|---|---|---|---|---|---|
| | ♂ | ♀ | ♂ (%) | ♀ (%) | ♂ (mo) | ♀ (mo) | ♂ (%) | ♀ (%) |
| Limited disease | 244 | 52 | 37 | 44 | 12.0 | 15.2 | 11 | 29 |
| Extensive disease | 402 | 63 | 17 | 27 | 8.7 | 10.2 | 3 | 9 |
| Karnofsky 50–70% | 128 | 25 | 11 | 20 | 5.9 | 9.0 | 2 | 0 |
| Karnofsky 80–100% | 519 | 90 | 28 | 39 | 10.4 | 13.4 | 7 | 24 |
| Non-smokers | 38 | 25 | 32 | 28 | 11.0 | 16.9 | 10 | 26 |
| Smokers | 608 | 90 | 24 | 37 | 9.7 | 11.8 | 6 | 17 |
| Weight loss | | | | | | | | |
| Less than 10% | 547 | 98 | 27 | 34 | 10.1 | 12.4 | 8 | 18 |
| More than 10% | 110 | 17 | 10 | 35 | 7.9 | 8.8 | 3 | 16 |
| Age | | | | | | | | |
| Less than 50 years | 131 | 26 | 27 | 46 | 10.5 | 15.2 | 5 | 18 |
| 50–59 years | 273 | 40 | 23 | 43 | 10.0 | 12.1 | 6 | 32 |
| 60 years and more | 253 | 49 | 25 | 22 | 9.1 | 9.3 | 7 | 8 |

median survival of 16.9 months and a 2-year survival rate for a proportion of 26% compared to a median survival of 11.0 months and a 2-year survival rate of 10% for non-smoking men. Due to the relatively small number of non-smokers, this difference failed to achieve statistical significance ($P = 0.065$, log rank test, stratified by extent of disease and performance status).

With smokers, the median survival rate (11.8 months *vs* 9.7 months) and the 2-year survival rate (17% *vs* 6%) were statistically significant favouring women with a *P*-value of 0.0025 (stratified log rank test). However, the quantity and duration of smoking slightly differed between male and female patients. The median cigarette consume for males was 40 packyears (one packyear = 20 cigarettes per day during 1 year) as opposed to 30 packyears for females. So far it has not been possible to affirm positively whether the difference in smoking quantity constitutes an additional prognostic variable due to the restricted data available about this problem.

*Weight loss prior to chemotherapy* As has been described before, the impact of weight loss prior to chemotherapy on survival was small. As expected, a better prognosis for female patients was found, both for patients with a weight loss of less than 10% and for patients with a weight loss of more than 10%. For further details see Table IV.

*Age* Most noteworthy were the results obtained with regard to age. Initially, three age categories were established with ranging from 18–49-year-olds, 50–59-, to 60–75-year-olds. While the prognosis for men was found to be the same regardless of age with a median survival of about 10.0 months and a 2-year survival rate for about 6% of the male patients, a striking difference in survival was seen for female patients of all three categories. Women aged less than 50 years (median survival 15.2 months, 2-year survival rate 18%) and women aged 50–59 years (median survival 12.1 months, 2-year survival rate 32%) achieved results superior to those of the older female population (median survival 9.3 months, 2-year survival rate 8%). The comparison of the survival curves of female and male patients was of statistical significance for the group of women aged less than 50 years ($P = 0.0057$) and for the ages 50–59 ($P = 0.0003$), whereas no difference was noted in the 59-plus-group ($P = 0.12$, stratified log rank test). The differing 2-year survival rates for females aged less than 50 years and 50–59 years are most likely an artefact which is due to the small patient number. The comparison of the survival curves of these two populations reveals no difference, whereas both curves are clearly superior to the survival of female patients older than 59 years. Therefore, the survival difference between younger and older women can be described in an appropriate way by using a two-stage classification which divides female patients into two groups, one including ages less than 60 years and one 60-plus. The comparison of the survival curves for both male and female patients in these two age groups are shown in Figures 2 and 3. Among the patients aged less than 60 years the advantage of the female sex is of high statistical significance with a *P*-value of 0.0001. For patients older than 59 years no difference in survival has been seen. In order to be able to compare the relative importance of the prognostic factors between males and females, a separate Cox proportional hazard model for prognostic variables in male and female patients was finally performed. The results of these analyses are summarised in Table V. The variable age was shown to have an important impact on the prognosis for women, but was irrelevant for men. In the female population, the four baseline characteristics performance status, extent of disease, history of smoking and age represented major prognostic factors, whereas in the male subpopulation only the three variables extent of disease, performance status and history of smoking had a significant impact on survival.

**Figure 2** Survival curve according to age (<60 years); (—) males, $n = 404$, median survival: 10.1 months; (----) females, $n = 65$, median survival: 13.3 months; $P = 0.0001$ (stratified log rank test).

**Figure 3** Survival curve according to age (older than 60 years); (—) males, $n = 249$, median survival: 9.1 months; (----) females, $n = 49$, median survival: 9.3 months; $P = 0.12$ (stratified log rank test).

**Table V** Results of Cox proportional hazard model* for females and males

| | | *Coefficient* | *Standard error* | *P-value* |
|---|---|---|---|---|
| Males | Performance status | 0.43 | 0.10 | 0.0001 |
| | Extent of disease | 0.59 | 0.09 | 0.0001 |
| | History of smoking | 0.37 | 0.17 | 0.032 |
| | Age | 0.06 | 0.08 | 0.46 |
| Females | Performance status | 0.77 | 0.27 | 0.0035 |
| | Extent of disease | 0.59 | 0.23 | 0.011 |
| | History of smoking | 0.60 | 0.26 | 0.024 |
| | Age | 0.49 | 0.21 | 0.017 |

*The columns of the table give the estimated regression coefficient for the corresponding dictotomous variable in the Cox model, its standard error and the *P*-value for the test of the hypothesis that the coefficient equals to zero.

The subgroup of patients with the most favourable outcome were limited stage female patients aged less than 60 years. They achieved a median survival of 16.4 months and a 3-year survival rate with a proportion of 32% compared to a median survival of 12.0 months and a 3-year survival rate of 7% for the corresponding male subgroup.

### Discussion

In the analysis of the data available about the prognostic value of baseline characteristics in SCLC, only the two variables 'extent of disease' and 'clinical performance status' have consistently constituted important predictors of survival (Morstyn *et al.*, 1984; Livingston *et al.*, 1978; Osterlind *et al.*, 1986; Souhami *et al.*, 1985; Kalter *et al.*, 1984; Vogelsang *et al.*, 1985; Ihde *et al.*, 1981; Rawson *et al.*, 1990). The influence of the sex on survival has been a matter of controversial discussion. Einhorn *et al.* (1978) and the two British analyses from Vincent *et al.* (1987) and Souhami *et al.* (1985) were unable to note any advantage for the female sex. Recently a large evaluation of prognostic factors in 3,873 patients with SCLC from ten centres of the United Kingdom reported by Rawson & Peto (1990) also failed to identify sex as a major prognostic factor. In the analysis of Ihde *et al.* (1981) from the NCI in Washington female patients achieved no superior survial to men either. This analysis, however, was based on 106 patients only, including 16 female ones. Recently, an updated evaluation of prognostic factors from the same institute dealing with 378 cases reported a statistically significant advantage of female patients over male ones, especially with regard to long-term survival (Dearing *et al.*, 1988). Several other large study groups have confirmed this observation and identified the sex as an independent prognostic factor in SCLC.

Maurer *et al.* (1981) from the CALGB reported 35% of 2-year survivals among female patients with limited disease and CR compared to 15% of 2-year survivals among male patients with limited disease and CR. Median survival, however, did not differ between men and women in limited stage nor in extensive stage. The analysis of Osterlind *et al.* (1986) from the Finsen Institute Copenhagen identified the female sex as a statistically significant favourable prognostic feature in limited stage with regard to 18 months disease-free survival. Out of 319 male and 124 female patients with limited stage, an 18 months disease-free survival rate was achieved by 11.9% of male and 16.9% of female patients. In extensive stage, no remarkable difference in prognosis was seen. The evaluation of Sagman *et al.* (1988) from Toronto also proclaimed the female gender to be a statistically significant predictor of long-term survival in 614 patients with SCLC. Recently, Spiegelman *et al.* (1989) from the CALGB published an analysis about prognostic factors of 1,521 patients and stated that the female gender was a significant predictor of improved survival in limited as well as in extensive stage patients.

The analysis presented here strongly supports these findings showing that women live significantly longer than men. This advantage of female over male patients was more pronounced in subgroups of patients with additional favourable prognostic features than in patients with additional adverse characteristics, and therefore, the sex has been an especialy important predictor for the probability of long-term survival.

Although most of the analyses by other investigators about prognostic variables are in accordance with our results, a few evaluations failed to demonstrate any difference. There is a multitude of factors which will have to be looked into when we are seeking to explain these controversial results and particular attention should be paid to the following aspects.

(a) First of all some analyses were based on small numbers of patients. The proportion of female patients in SCLC is only about 15–20%, so that only large trials including a sufficient number of women allow statistical evaluation.

(b) The prognostic value of the sex seems to be more pronounced in subgroups of patients with additional favourable prognostic features than in subgroups with adverse characteristics. This suggestion is supported by several of the aforementioned investigations where advantageous prognoses were proven for female patients especially in limited stage, whereas no difference in extensive stage patients was seen (Osterlind *et al.*, 1986; Vincent *et al.*, 1987).

(c) The prognostic value of the sex seems to be especially important for long-term survival. This observation complements the finding that the sex is an important prognostic factor in subgroups of patients with additional favourable prognostic features. Only these people live long enough to demonstrate an impact of a specific variable on late phases of the survival curve, whereas patients with additional adverse prognostic factors die early in the course of treatment irrespective of the presence of a single favourable parameter. In accordance with our results, the analysis of the Toronto group, as well as the Danish group and the CALGB identified the sex as an important predictor for long-term survival. On the other hand, Souhami *et al.* (1990) noticed no major impact on the survival of SCLC patients in the United Kingdom.

(d) At this moment in time a sufficient explanation for these controversial results is not available. Perhaps special regional factors and environmental stresses or differences in smoking behaviour have to be regarded as additional prognostic factors. However, the results of our analyses point to the suggestion that the relationship between the patient's sex and age has to be considered as an important predictor for survival. The advantage of female patients seems to be restricted to younger ones. When analysing our data, we noticed the striking statistical significance of the advantage of female patients aged less than 60 years over male ones, whereas no difference in survival was seen for patients in the 60-plus group.

This relationship of sex and age has not been considered in any other evaluation. If predominantly older females had been recruited in these trials, the differences in prognosis might have been covered. However, in spite of the striking superiority of the female sex in our data set, we have to admit that the number of female patients was relatively small, so that our findings about the relationshiop between the patient's sex and age will have to be confirmed with the help of a larger set of patients.

The reasons for the superiority of the female sex are still not completely known. It is obvious that women have a better prognosis than men in a large variety of malignant diseases. In SCLC several aspects have been suggested as contributors to this advantage. Osterlind *et al.* (1986) suggested that female patients as opposed to male ones may receive a rather more aggressive chemotherapy. In their analysis leukocyte nadirs were lower in women than in men which is likely to favour this hypothesis. Spiegelman *et al.* (1989) also performed such an evaluation but this group noticed no difference in myelotoxicity, performance of treatment and side effects between male and female patients. When analysing our own data, we, too, were unable to confirm the suggestion by Osterlind *et al.* (1986) leukocyte nadirs and leukocyte values prior to each cycle of chemotherapy did not differ between both groups. Furthermore, clinical side effects according to the WHO criteria occurred in nearly the same frequencies in both, female and male patients. The performance of treatment was comparable, too, with a slightly higher number of cycles of chemotherapy and irradiation given to men. From these data we conclude that the advantage of the female sex was not due to differences in the performance of treatment.

A second aspect which has to be considered is the different history of smoking of female and male patients. The proportion of non-smokers was higher in women than in men, and even the smoking women had less tobacco consume than the smoking men. The history of smoking seems to be a further independent prognostic variable with favourable impact on survival for non-smokers. But even when the history of smoking was included into the multivariate test systems, the sex still remains an independent prognostic factor. However, as for the analysis of the relationship of sex and age, we have to admit that our patient population and especially the number of female ones and non-smokers are too small for a definitive evaluation.

As to the observation that the better prognosis of females was restricted to the younger population we suggested that sexual hormones may play a role in the regulation of tumour growth and should thus be taken into consideration as a possible reason for the advantage of the female gender. On average, in the age of 45 years estrogen and progesteron levels begin to decrease and reach the lowest values in the middle of the 5th decade of life. Therefore, women older than 55 years have low female sexual hormone levels and relatively high androgen levels. On the basis of these facts we suggested that either female sexual hormones like estrogen and progesteron may have a protective function or male sexual hormones like androgen may force tumour growth in SCLC.

In order to test this hypothesis, the influence of sexual hormones on cell proliferation in permanent human small cell lung cancer cell lines has been investigated in our laboratories. Maasberg *et al.* (1989) found that 8/13 cell lines had androgen receptors. The application of testosterone resulted in a 3-fold increase of cell proliferation in the cloning assay, whereas estrogen had no influence on tumour growth. Subsequently an anti-androgen was added to this test system and resulted in a complete compensation of the androgen effects. The stimulation by androgen was confirmed by thymidine incorporation assay. Using this method, dehydro-testosterone was clearly more effective than testosterone suggesting that the stimulation of tumour growth is predominantly due to this metabolite. Dehydro-testosterone is built by the enzyme 5a-reductase at the level of the cell membrane. 5a-reductase activity was detected in nearly all cell lines indicating that human small cell lung cancer cells are able to convert testosterone in dehydro-testosterone. These *in vitro* experiments showing an enhancement of cell proliferation by androgens hint that sexual hormones may play a role in the regulation of tumour growth in SCLC.

This suggestion will need further confirmation in experimental and clinical studies. However, from these results a new treatment approach in SCLC may be derived which focuses on the decrease of androgen levels in males by means of anti-androgens and LH-RH-agonists. We will test this strategy and hope to establish a new treatment modality which may overcome the unique results of chemotherapy in SCLC during the last decade.

The authors wish to thank Mrs C. Braun for assisting in data collection and careful preparation of this manuscript. The excellent collaboration of the Biostatistics and Data Centre (ZMBT), University of Heidelberg, in collection and analyses of the data presented here is also acknowledged.

The trials were supported by the Federal Ministry of Research and Technology (Bundesministerium für Forschung und Technologie; BMFT).

### References

COX, D.R. (1972) Regression models and life tables. *J.R. Stat. Soc. Serv.*, **34**, 187–220.

DEARING, M.P., STEINBERG, S.M., PHELPS, R. & 4 others (1988). Women small cell lung cancer (SCLC) patients live longer than males. *Proc. Am. Soc. Clin. Oncol.*, **7**, 198, abstract.

EINHORN, L.H., BOND, W.H, HORNBACK, N. & JOE. B.T. (1987). Long-term results in combined modality treatment of small cell carcinoma of the lung. *Semin. Oncol.*, **5**, 309–313.

HAVEMANN, K., WOLF, M., HOLLE, R. & 11 others (1987*a*). Alternating versus sequential chemotherapy in small cell lung cancer. *Cancer*, **59**, 1072–1082.

HAVEMANN, K., WOLF, M., HOLLE, R. & 5 others (1987*b*). Cyclic alternating versus response-orientated alternating chemotherapy in small cell lung cancer (SCLC). *Proc. Eur. Conf. Clin. Oncol.*, **9**.

IHDE, D.C., MAKUCH, R.W., CARNEY, D.N. & 4 others (1981). Prognostic implications of stage of disease and sites of metastases in patients with small cell carcinoma of the lung treated with intensive combination chemotherapy. *Am. Rev. Respir. Dis.*, **123**, 500–507.

KALTER, S., FARHA, P., CARR, D.T., JEFFRIES, D., LEE, J.S. & VALDIVIESO, M. (1984). Long-term survivors with small cell lung cancer: The M.D. Anderson experience from 1972–1980. *Proc. Am. Soc. Clin. Oncol.*, **3**, 229, abstract.

LIVINGSTON, R.B., MOORE, T.N., HEILBRUN, L. & 4 others (1978). Small cell carcinoma of the lung: combined chemotherapy and radiation. *Ann. Int. Med.*, **88**, 194–199.

MAASBERG, M., JAQUES, G., ROTSCH, M., ENDERLE-SCHMIDT, U., WEEHLE, R. & HAVEMANN, K. (1989). Androgen receptors, androgen dependent proliferation, and 5-reductase activity of small cell lung cancer cell lines. *Int. J. Cancer*, **43**, 685–691.

MAURER, L.H. & PAJAK, T.F. (1981). Prognostic factors in small cell carcinoma of the lung: a cancer and leukemia group B study. *Cancer Treat. Rep.*, **65**, 767–774.

MINNA, J.D., HIGGINS, G.A. & GLATSTEIN, E.J. (1985). Cancer of the lung. Devita, V.T., Hellman, S., Rosenberg, S.A. (eds). *Cancer: Principles and practice of oncology*. Lippincott: Philadelphia, 507–597.

MORSTYN, G., IHDE, D.C., LICHTER, A.S. & 4 others (1984). Small cell lung cancer 1973–1983: Early progress and recent obstacles. *Int. J. Radiat. Oncol. Biol. Phys.*, **10**, 515–539.

OSTERLIND, K., HANSEN, H.H., HANSEN, M., DOMBERNOWSKY, P. & ANDERSEN, P.K. (1986). Long-term disease-free survival in small cell carcinoma of the lung: a study of clinical determinants. *J. Clin. Oncol.*, **4**, 1307–1313.

OSTERLIND, K. (1986). Prognostic factors in small cell lung cancer: an analysis of 874 consecutive patients. In: Hansen, H.H., (ed.) *Lung Cancer: Basic and clinical aspects*. Martinus Nijhoff Publishers, Boston, 129–152.

PETO, R., PIKE, M.C., ARMITAGE, P. & 5 others (1977). Design and analyses of randomized clinical trials requiring prolonged observation of each patient. II Analyses and examples. *Br. J. Cancer*, **35**, 1–39.

RAWSON, N.S.B. & PETO, J. (1990). An overview of prognostic factors in small cell lung cancer. *Br. J. Cancer*, **61**, 597–604.

SAGMAN, U., FELD, R., DE BOER, G. & 6 others (1988). Long-term survival of patients (pts) with small cell lung cancer (SCLC) – the Toronto experience (1976–1986). *ASCO Proc.*, **7**, 203, abstr. 786.

SOUHAMI, R.L., BRADBURY, I., GEDDES, D.M., SPIRO, S.G., HARPER, P.G. & TOBIAS, J.S. (1985). Prognostic significance of laboratory parameters measured at diagnosis in small cell carcinoma of the lung. *Cancer Res.*, **45**, 2878–2882.

SOUHAMI, R.L. & LAW, K. (1990). Longevity in small cell lung cancer. *Br. J. Cancer*, **61**, 584–589.

SPIEGELMAN, D., MAURER, L.H., WARE, J.H. & 6 others (1989). Prognostic factors in small-cell carcinoma of the lung: An analysis of 1,521 patients. *J. Clin. Oncol.*, **7**, 344–354.

VINCENT, M.D., ASHLEY, J.E. & SMITH, I.A. (1987). Prognostic factors in small cell lung cancer: A simple prognostic index is better than conventional staging. *Eur. J. Cancer Clin. Oncol.*, **23**, 1589–1599.

VOGELSANG, G.B., ABELOFF, M.D., ETTINGER, D.S. & BOOKER, S.U. (1985). Long-term survivors of small cell carcinoma of the lung. *Am. J. Med.*, **79**, 49–56.

WOLF, M., HAVEMANN, K., HOLLE, R. & 12 others (1987). Cisplatinum/etoposide (PE) versus ifosfamide/etoposide (IE) combination chemotherapy in small cell lung cancer (SCLC). A multicenter German randomized trial. *J. Clin. Oncol.*, **5**, 1880–1889.


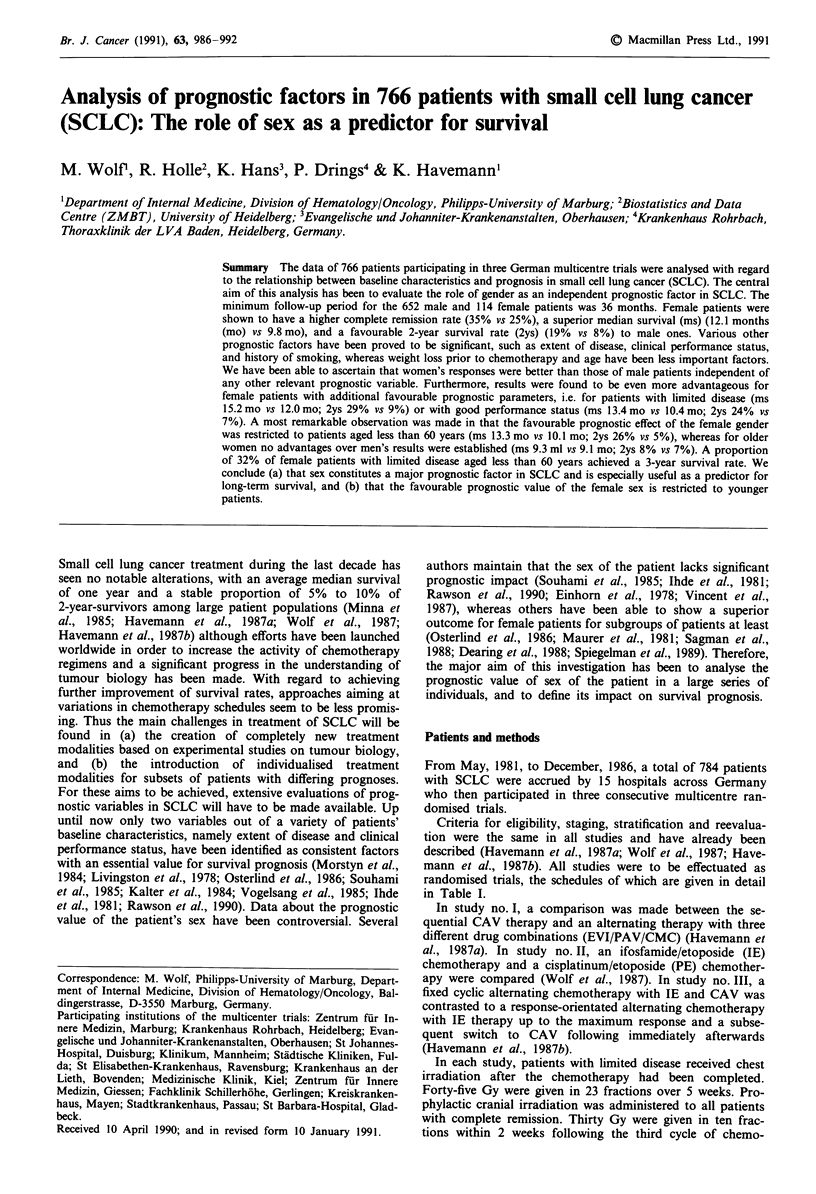

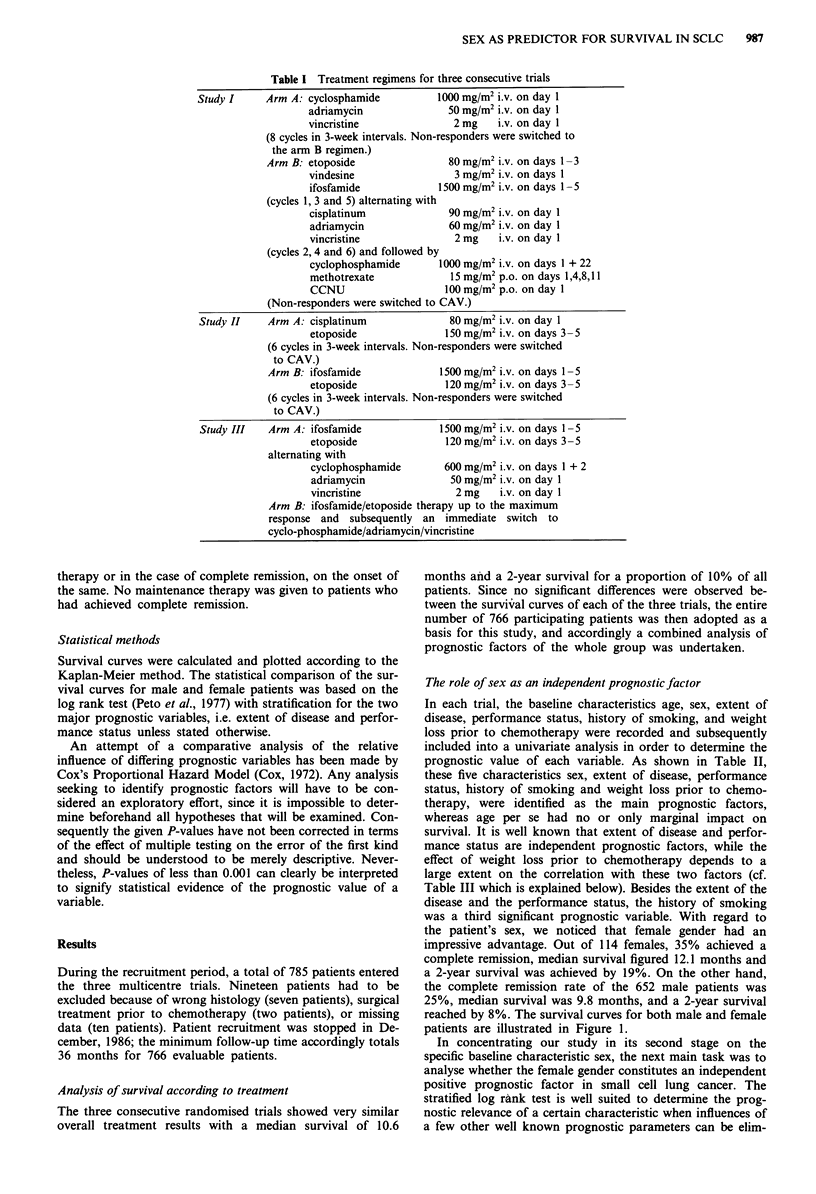

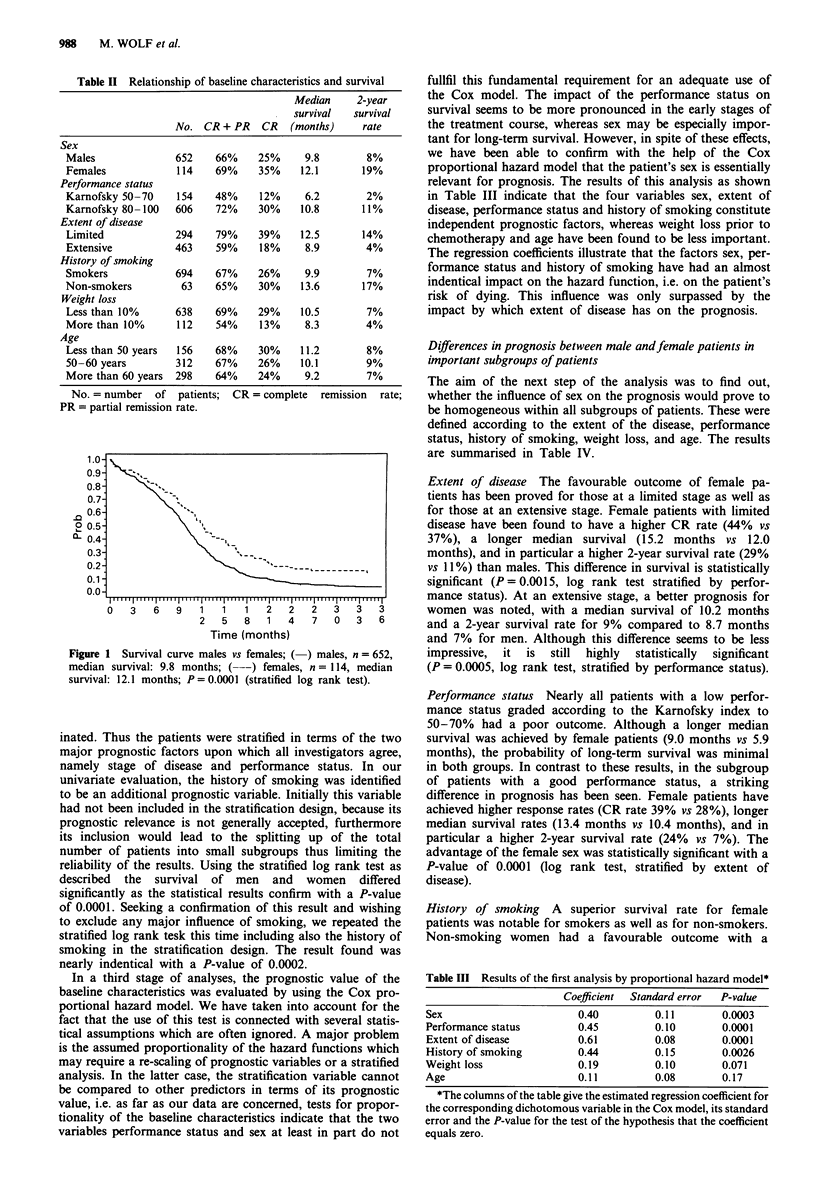

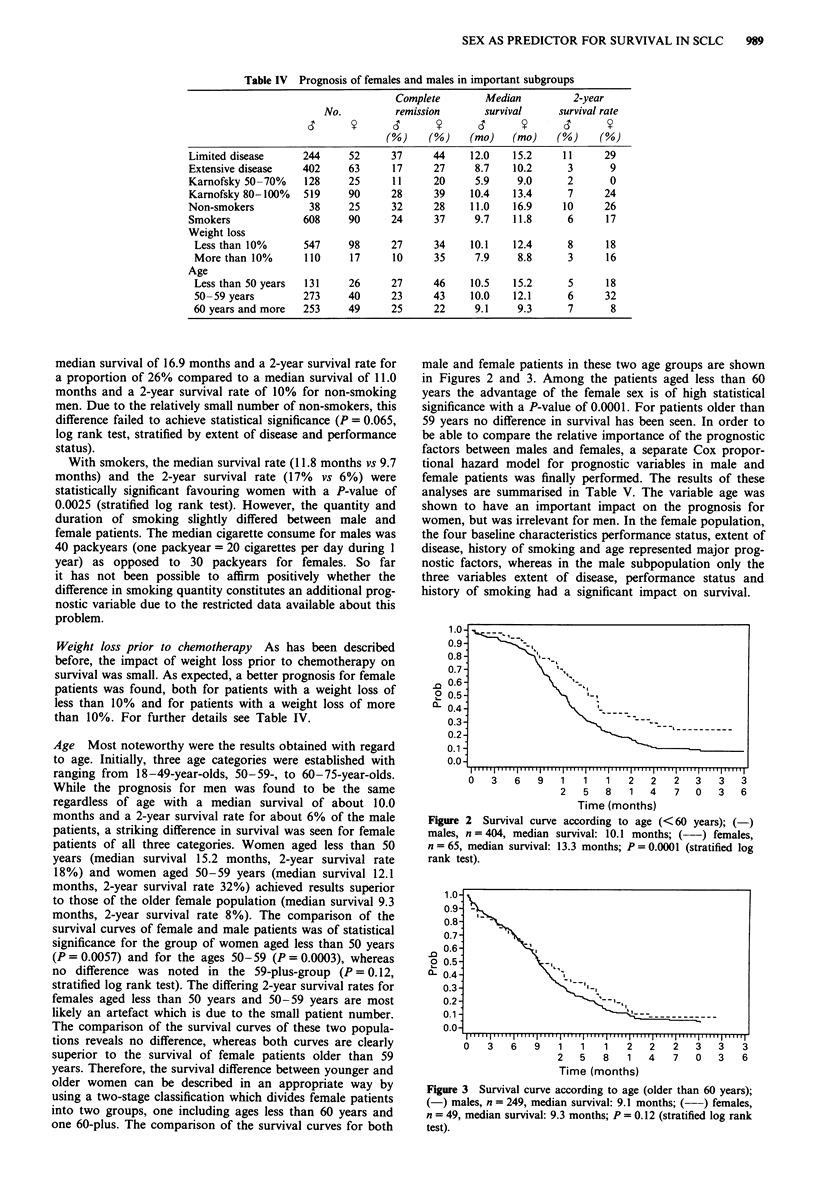

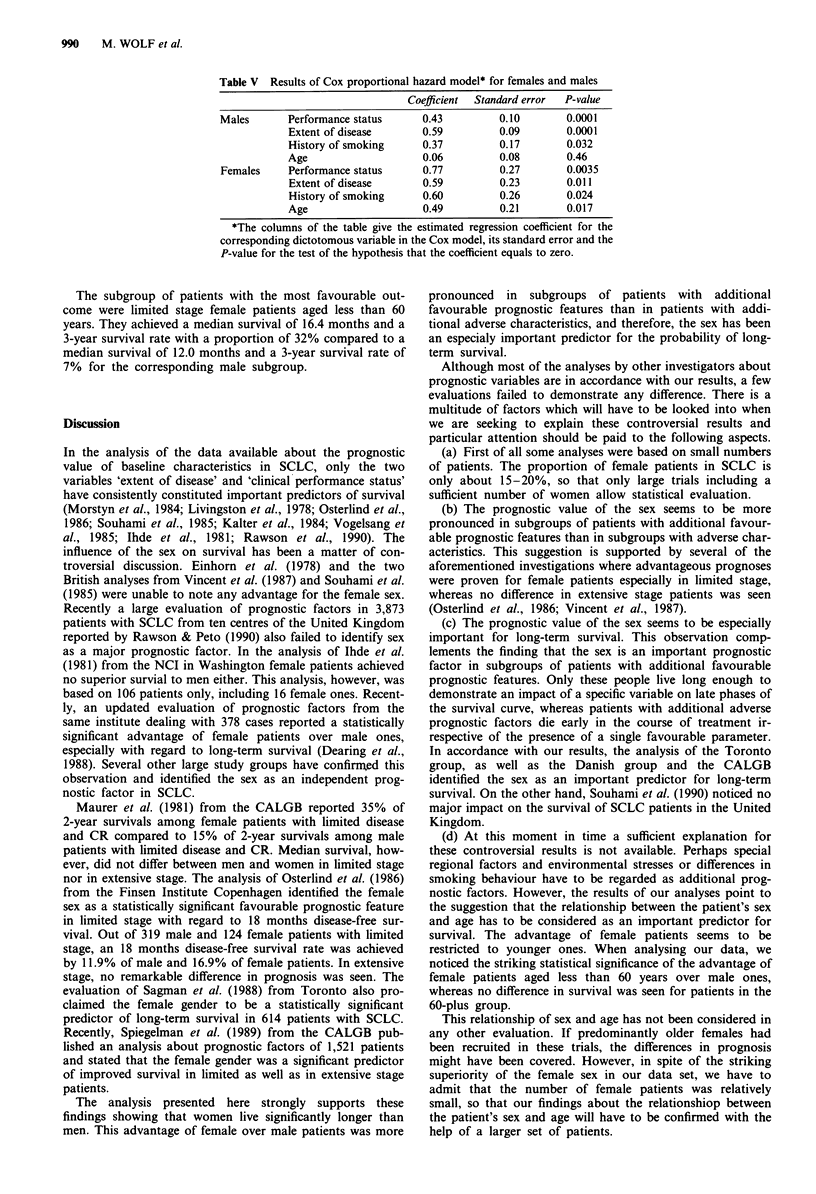

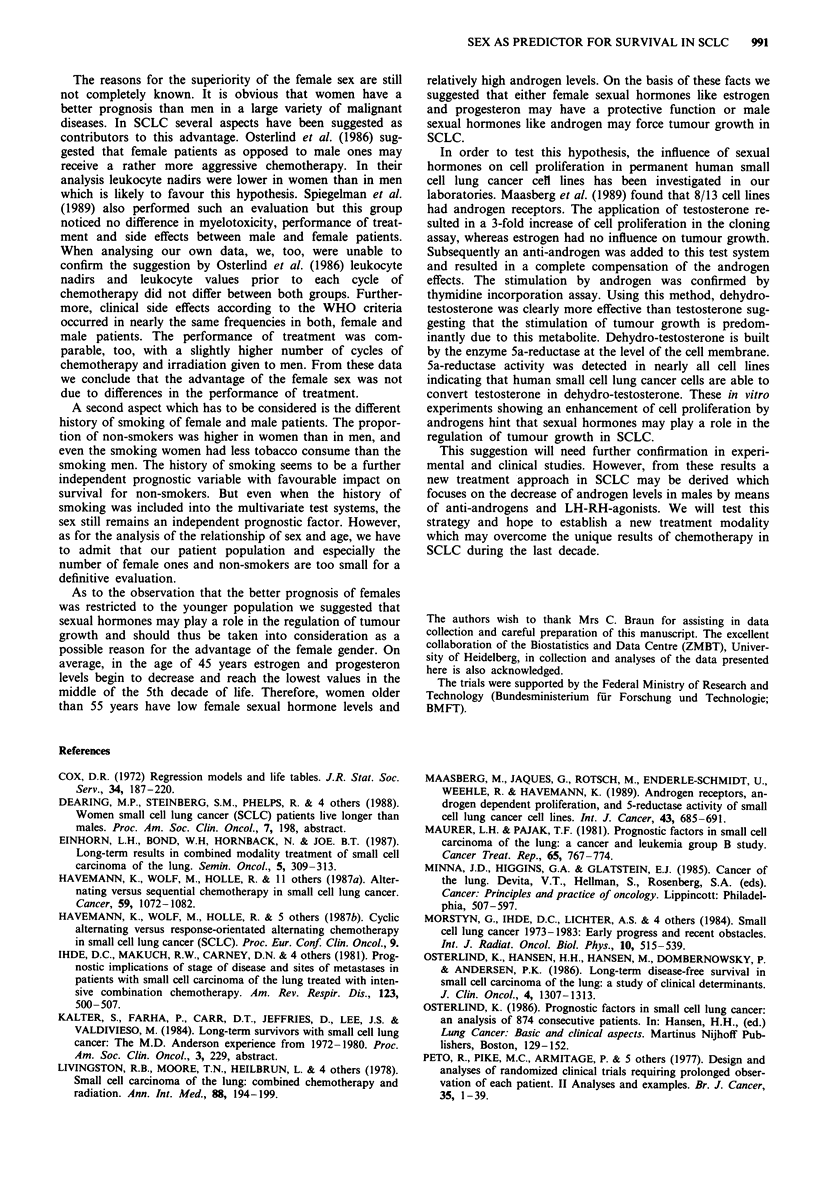

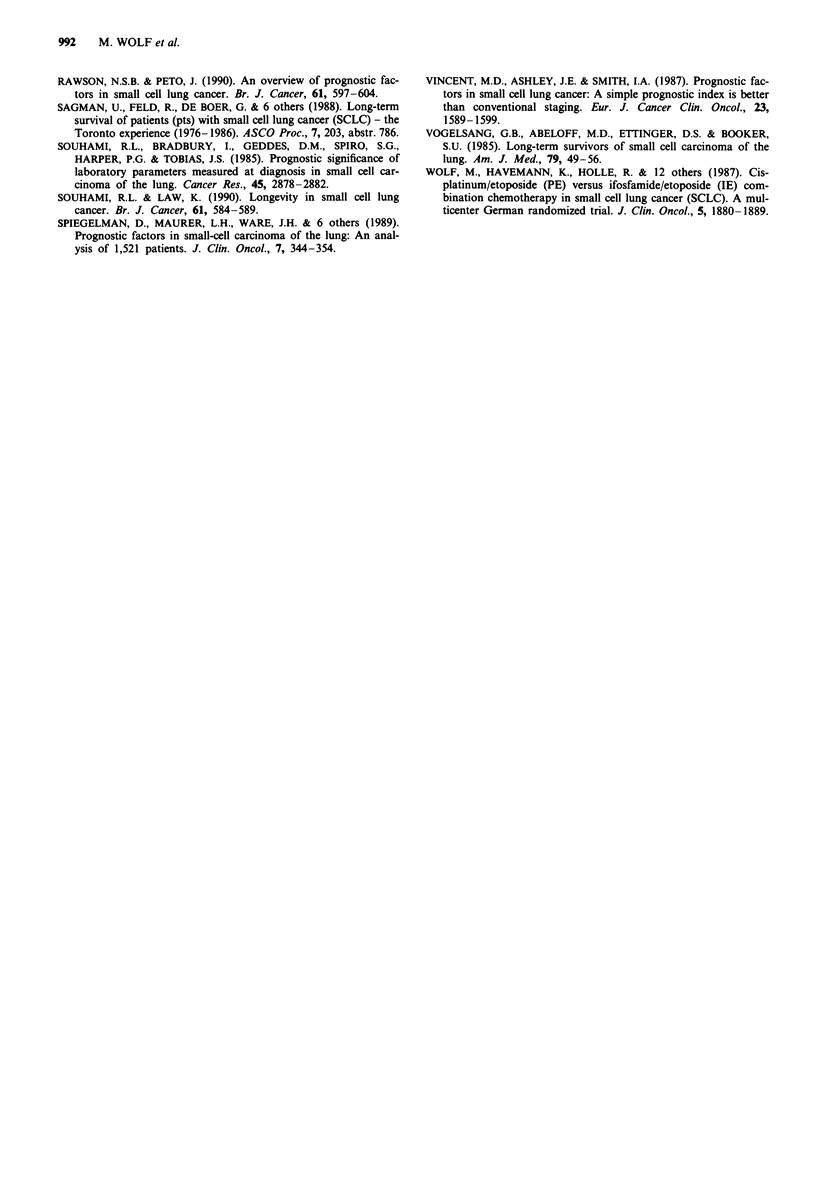


## References

[OCR_00865] Einhorn L. H., Bond W. H., Hornback N., Joe B. T. (1978). Long-term results in combined-modality treatment of small cell carcinoma of the lung.. Semin Oncol.

[OCR_00870] Havemann K., Wolf M., Holle R., Gropp C., Drings P., Manke H. G., Hans K., Schroeder M., Heim M., Victor N. (1987). Alternating versus sequential chemotherapy in small cell lung cancer. A randomized German multicenter trial.. Cancer.

[OCR_00881] Ihde D. C., Makuch R. W., Carney D. N., Bunn P. A., Cohen M. H., Matthews M. J., Minna J. D. (1981). Prognostic implications of stage of disease and sites of metastases in patients with small cell carcinoma of the lung treated with intensive combination chemotherapy.. Am Rev Respir Dis.

[OCR_00892] Livingston R. B., Moore T. N., Heilbrun L., Bottomley R., Lehane D., Rivkin S. E., Thigpen T. (1978). Small-cell carcinoma of the lung: combined chemotherapy and radiation: a Southwest Oncology Group study.. Ann Intern Med.

[OCR_00897] Maasberg M., Rotsch M., Jaques G., Enderle-Schmidt U., Weehle R., Havemann K. (1989). Androgen receptors, androgen-dependent proliferation, and 5 alpha-reductase activity of small-cell lung cancer cell lines.. Int J Cancer.

[OCR_00903] Maurer L. H., Pajak T. F. (1981). Prognostic factors in small cell carcinoma of the lung: a cancer and leukemia group B study.. Cancer Treat Rep.

[OCR_00914] Morstyn G., Ihde D. C., Lichter A. S., Bunn P. A., Carney D. N., Glatstein E., Minna J. D. (1984). Small cell lung cancer 1973-1983: early progress and recent obstacles.. Int J Radiat Oncol Biol Phys.

[OCR_00919] Osterlind K., Hansen H. H., Hansen M., Dombernowsky P., Andersen P. K. (1986). Long-term disease-free survival in small-cell carcinoma of the lung: a study of clinical determinants.. J Clin Oncol.

[OCR_00931] Peto R., Pike M. C., Armitage P., Breslow N. E., Cox D. R., Howard S. V., Mantel N., McPherson K., Peto J., Smith P. G. (1977). Design and analysis of randomized clinical trials requiring prolonged observation of each patient. II. analysis and examples.. Br J Cancer.

[OCR_00939] Rawson N. S., Peto J. (1990). An overview of prognostic factors in small cell lung cancer. A report from the Subcommittee for the Management of Lung Cancer of the United Kingdom Coordinating Committee on Cancer Research.. Br J Cancer.

[OCR_00947] Souhami R. L., Bradbury I., Geddes D. M., Spiro S. G., Harper P. G., Tobias J. S. (1985). Prognostic significance of laboratory parameters measured at diagnosis in small cell carcinoma of the lung.. Cancer Res.

[OCR_00953] Souhami R. L., Law K. (1990). Longevity in small cell lung cancer. A report to the Lung Cancer Subcommittee of the United Kingdom Coordinating Committee for Cancer Research.. Br J Cancer.

[OCR_00957] Spiegelman D., Maurer L. H., Ware J. H., Perry M. C., Chahinian A. P., Comis R., Eaton W., Zimmer B., Green M. (1989). Prognostic factors in small-cell carcinoma of the lung: an analysis of 1,521 patients.. J Clin Oncol.

[OCR_00962] Vincent M. D., Ashley S. E., Smith I. E. (1987). Prognostic factors in small cell lung cancer: a simple prognostic index is better than conventional staging.. Eur J Cancer Clin Oncol.

[OCR_00968] Vogelsang G. B., Abeloff M. D., Ettinger D. S., Booker S. V. (1985). Long-term survivors of small cell carcinoma of the lung.. Am J Med.

[OCR_00973] Wolf M., Havemann K., Holle R., Gropp C., Drings P., Hans K., Schroeder M., Heim M., Dommes M., Mende S. (1987). Cisplatin/etoposide versus ifosfamide/etoposide combination chemotherapy in small-cell lung cancer: a multicenter German randomized trial.. J Clin Oncol.

